# GAS2-like proteins mediate communication between microtubules and actin through interactions with end-binding proteins

**DOI:** 10.1242/jcs.140558

**Published:** 2014-06-15

**Authors:** Matthew J. Stroud, Alicja Nazgiewicz, Edward A. McKenzie, Yisu Wang, Richard A. Kammerer, Christoph Ballestrem

**Affiliations:** 1 Wellcome Trust Centre for Cell-Matrix Research, Faculty of Life Sciences, University of Manchester, Manchester M13 9PT, UK; 2 Manchester Institute of Biotechnology, Faculty of Life Sciences, 131 Princess Street, Manchester M1 7DN, UK; 3 Laboratory of Biomolecular Research, OFLC 106, Paul Scherrer Institut, 5232 Villigen PSI, Switzerland

**Keywords:** GAS2 family, GAS2-like 1, GAS2-like 2, GAS2-like 3, End-binding protein, Microtubule, Actin, MT-tip localising signal, MtLS

## Abstract

Crosstalk between the microtubule (MT) and actin cytoskeletons is fundamental to many cellular processes including cell polarisation and cell motility. Previous work has shown that members of the growth-arrest-specific 2 (GAS2) family mediate the crosstalk between filamentous actin (F-actin) and MTs, but the molecular basis of this process remained unclear. By using fluorescence microscopy, we demonstrate that three members of this family, GAS2-like 1, GAS2-like 2 and GAS2-like 3 (G2L1, G2L2 and G2L3, also known as GAS2L1, GAS2L2 and GAS2L3, respectively) are differentially involved in mediating the crosstalk between F-actin and MTs. Although all localise to actin and MTs, only the exogenous expression of G2L1 and G2L2 influenced MT stability, dynamics and guidance along actin stress fibres. Biochemical analysis and live-cell imaging revealed that their functions are largely due to the association of these proteins with MT plus-end-binding proteins that bind to SxIP or SxLP motifs located at G2L C-termini. Our findings lead to a model in which end-binding (EB) proteins play a key role in mediating actin–MT crosstalk.

## INTRODUCTION

Crosstalk between microtubules (MTs) and actin is essential for normal cellular function. The most prominent candidates for this role are the spectraplakins, proteins that crosslink MTs and actin (Leung et al., 1999; Kodama et al., 2003; Wu et al., 2008; Prokop et al., 2013). Various medical conditions and developmental defects arise as a result of mutations in genes encoding spectraplakins, including mental retardation, cancer and chronic skin blistering (Sonnenberg and Liem, 2007). Spectraplakins are multi-domain proteins with binding sites for filamentous actin (F-actin) in their calponin homology (CH) domains, and for MTs in their C-terminal regions. Mice lacking MT-actin crosslinking factor 1 (MACF1), a spectraplakin family member, show embryonic lethality, and dystonin mutant patients or mice suffer from sensory neuropathy, which has been correlated with instability and aberrant organisation of MTs (Bernier et al., 1995; Brown et al., 1995; Guo et al., 1995; Goryunov et al., 2007). Cells without MACF1 display defects in the guidance of MTs along actin stress fibres and in cellular polarisation (Kodama et al., 2003). Expression of a mini-version of MACF1, consisting of the actin- and MT-binding domains only, was sufficient to rescue these perturbations (Kodama et al., 2003), implying that these are key functional domains of spectraplakins.

Members of the growth-arrest-specific 2 (GAS2) family resemble spectraplakins as they possess both actin- and MT-binding regions, but they lack plakin domains. The GAS2 family consists of four members: GAS2, GAS2-like 1, GAS2-like 2 and GAS2-like 3 (G2L1, G2L2 and G2L3, also known as GAS2L1, GAS2L2 and GAS2L3, respectively) (Schneider et al., 1988; Goriounov et al., 2003; Stroud et al., 2011).

GAS2 is widely expressed in human tissues (Collavin et al., 1998). It has been found to play a role in apoptosis (Brancolini et al., 1995; Lee et al., 1999; Sgorbissa et al., 1999) and to inhibit cell division in *Xenopus* embryos (Zhang et al., 2011). G2L1 is expressed in testis and brain and is involved in inhibiting the growth of red blood cells downstream of thyroid receptor signalling (Goriounov et al., 2003; Gamper et al., 2009). G2L2 is exclusively expressed in skeletal muscle, but little is known about its function (Goriounov et al., 2003). G2L3 is found in many cell types and we have previously demonstrated that it binds to actin and MTs (Stroud et al., 2011). It is also specifically upregulated during mitosis and contributes to cell cycle regulation (Wolter et al., 2012). Knockdown of G2L3 in human BJ fibroblasts and HCT116 cells resulted in aneuploidy, implying that deregulation of G2L3 might play a role in tumorigenesis (Wolter et al., 2012).

Although a potential function of the GAS2 family in the crosstalk between actin and MTs has been proposed, little is known about how it is mediated (Goriounov et al., 2003). All GAS2 family members contain a CH domain (a putative active-binding site) and a GAS2-related (GAR) domain (a putative MT-binding domain), but only the GAS2-like proteins contain a larger unstructured C-terminus. Further examination of the C-termini of G2L1, G2L2 and G2L3 proteins has revealed that, like spectraplakins, they contain evolutionarily-conserved MT-tip localisation signals (MtLSs) comprising the amino acid sequence Ser/Thr-Xaa-Ile/Leu-P (or SxIP motifs), necessary to interact with MT plus-end-binding (EB) proteins (Honnappa et al., 2009). G2L1 and G2L2 have recently been identified in a proteome-wide screen for EB-binding proteins (Jiang et al., 2012), but it was not clear whether these sites are functionally relevant or what role they might have.

In the present study, we aimed to gain mechanistic insight into the role of GAS2 family members in cells. We found that whereas full-length GAS2 localised exclusively to actin stress fibres, G2L1, G2L2 and G2L3 colocalised with both actin stress fibres and MTs, and contributed to different levels of actin–MT co-alignment. The identification of EB-binding motifs in the C-termini of G2L proteins led to our hypothesis that EB binding might play an important role in the cytoskeletal crosstalk. This was indeed the case for G2L1 and G2L2, which influenced not only MT guidance along actin stress fibres, but also MT dynamics and stability.

## RESULTS

### Expression of G2L1 and G2L2 induce actin–microtubule co-alignment

**Fig. 1.**
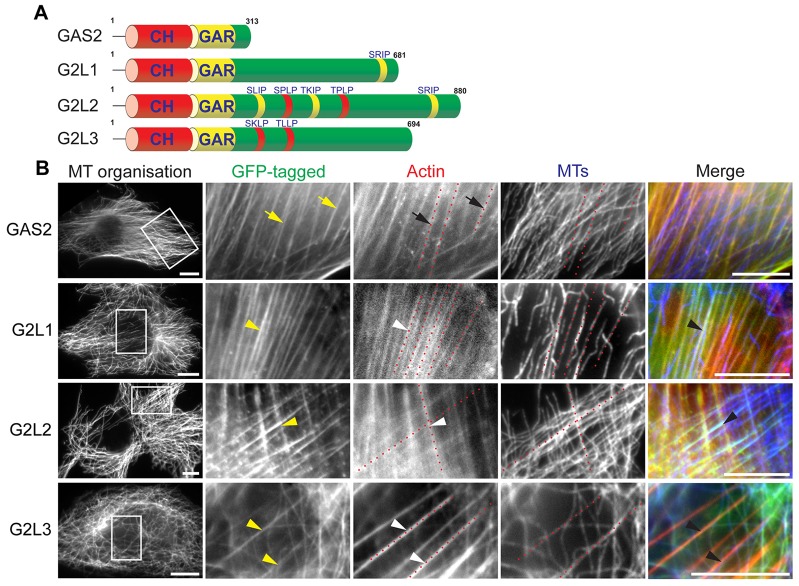
**Subcellular localisation of the GAS2 family members.** (A) Schematic representation of members of the GAS2 family. The calponin homology (CH) and GAS2-related (GAR) domains are depicted in red and yellow, respectively, and the number of amino acids for each family member is noted above the C-termini. The sequences of the MtLSs are denoted above their respective C-termini, SxIP motifs are yellow, S/TxLP motifs are red. Note that the β-isoforms of G2L1 and G2L2 are depicted. (B) NIH3T3 cells expressing the indicated constructs were fixed and stained for MTs and actin. Dotted red lines indicate where the actin cytoskeleton is in respect to the MTs. Note that GAS2 (yellow arrows) localised exclusively to the actin cytoskeleton (black arrows), whereas G2L1, G2L2 and G2L3 (yellow arrowheads) localised to both MTs (black arrowheads) and actin-rich structures (white arrowheads). Scale bars: 10 µm.

To compare the subcellular localisation of the GAS2 family of proteins (Fig. 1A) we transiently expressed them in NIH3T3 fibroblasts. GAS2, G2L1 and G2L2 localised predominantly to actin stress fibres. In the case of GAS2, MTs seemed to localise independently of actin, whereas for G2L1 and G2L2 they showed high incidence of co-alignment with stress fibres, suggesting a role for these two proteins in MT-actin crosslinking. Despite the localisation of G2L3 to actin and MTs we found little co-alignment of the two (Fig. 1B).

### MtLSs in G2L proteins are essential for microtubule plus-end localisation

**Fig. 2.**
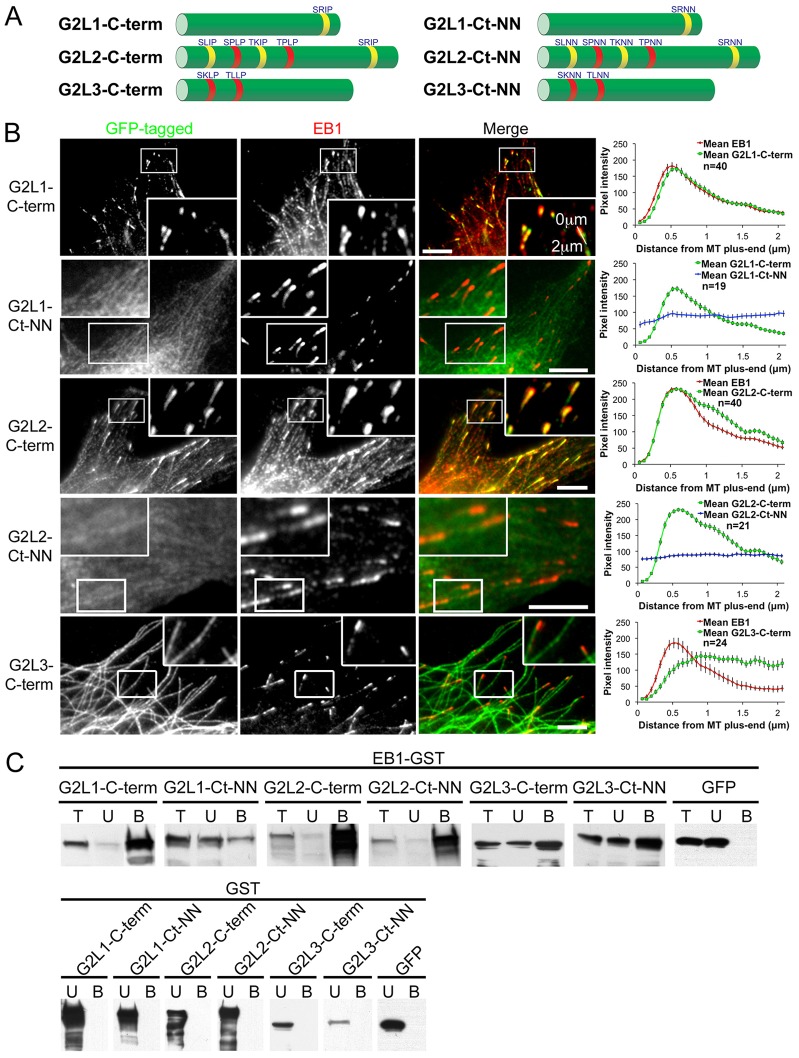
**MtLSs in GAS2-like proteins are essential for plus-end localisation and augment their binding to EB1.** (A) Schematic representation of the C-termini of the GAS2-like proteins used in this study, with their MtLSs indicated (left panel), and their respective mutants in which the isoleucine/leucine-proline (I/L-P) residues have been mutated to two asparagine residues (NN) (right panel). Note that G2L1 contains one MtLS, and G2L2 and G2L3 contain five and two MtLSs, respectively. The sequences of the MtLSs are denoted above their respective C-termini, SxIP motifs are yellow, S/TxLP motifs are red. (B) NIH3T3 cells expressing the indicated constructs were fixed and stained for EB1. Lines were drawn from the end of the MT plus-end through the MT towards the cell interior (as indicated in G2L1-C-term inset), and pixel intensities were recorded for both EB1 and the indicated construct (right panel). Note that the G2L1-C-term and G2L2-C-term colocalised with EB1 decorating MT plus-ends. Conversely, G2L1-Ct-NN and G2L2-Ct-NN mutants localised along the MT lattice. G2L3-C-term localises along the microtubule lattice. Rectangular regions of interest are enlarged in each panel. The number of MT plus-ends scored is given; results are mean±s.e.m. Scale bars: 5 µm. (C) COS1 cells expressing the indicated GFP-tagged construct were lysed and incubated with GST–EB1 (top panel) or GST (control)-coated glutathione beads (lower panel). The total cell lysate (T), unbound (U) and bound (B) fractions were taken and immunoblotted using an anti-GFP antibody. Note that G2L1-C-term, G2L2-C-term and G2L3-C-term all bind strongly to EB1, in comparison to their respective mutants, which bind more weakly. GST-coated control beads do not interact with any of the G2L family members, as they are not found in the bound fractions.

Previous studies have suggested that the C-termini of G2L proteins are crucial for MT binding (Goriounov et al., 2003; Stroud et al., 2011; Jiang et al., 2012). This was supported by our previous observations that GAS2, the only member of the family without an extended C-terminus, localises only to actin stress fibres, and that the other members lacking the C-terminal tail localise exclusively to stress fibres (Goriounov et al., 2003; Stroud et al., 2011). To provide more detailed understanding of G2L-protein–MT interactions, we analysed the sequences of their C-termini and revealed that all of them contained putative binding sites for EB proteins. G2L1 had one potential MtLS, G2L2 had five and G2L3 had two (Fig. 2A). The single MtLS in G2L1, and the last MtLS in G2L2 are well conserved in both mice and zebrafish.

To investigate the role of the C-termini of G2L proteins and their functional significance to EB proteins, we expressed the isolated C-termini fused to GFP in NIH3T3 cells, fixed the cells and then stained for EB1 (also known as MAPRE1). The results showed that both G2L1-C-term and G2L2-C-term (Fig. 2A) decorated the MT plus-ends, where endogenous EBs reside. Line-tracing analysis of the MT plus-end revealed that the C-termini of both G2L1 and G2L2 colocalised with EB1, consistent with the idea that they interact with EB1 (Fig. 2B). Time-lapse recordings demonstrated that G2L1 and G2L2 C-termini robustly track the tip of (tip-track) MT plus-ends in a similar manner to the EB proteins (supplementary material Movie 1). Interestingly, G2L3-C-term did not tip track MTs but decorated the whole MT shaft (Fig. 2B).

To examine the function of the potential EB-binding sites in G2L proteins we mutated the IP or LP residues in the MtLSs to two asparagine residues (NN) (Fig. 2A). These mutations abolished MT-end localisation and MT-plus-end tracking of the C-termini of G2L1 and G2L2. The C-terminus of G2L3 remained at the MT shaft despite mutating its potential EB-binding sites (Fig. 2B), suggesting that there are other motifs in its C-terminus that mediate MT binding.

To confirm the interaction of EBs with the C-termini of the G2L proteins, we performed GST-pulldown assays. For these experiments, we expressed G2L C-termini in COS1 cells and used EB1 to pull down the three G2L C-termini (Fig. 2C). All three G2L C-termini were present in the bound fraction demonstrating their interaction with EB1. This was dramatically reduced in the MtLSs mutant constructs of G2L1 and G2L2 C-termini, but not for the mutation of G2L3 C-terminus.

Taken together, these data suggest that the C-termini of G2L1 and G2L2 proteins tip-track MT plus-ends by interacting with EB proteins through their MtLSs.

### G2L1 and G2L2 located at actin stress fibres bind EB proteins

Because of our observation that overexpression of G2L1 and G2L2 increased co-alignment of MTs with F-actin stress fibres, we wondered whether full-length G2L proteins affected MT dynamics, and to what extent this depended on the interaction between G2L proteins and EBs.

To investigate MT growth rates, G2L proteins were co-expressed together with tdTomato-tagged EB3 (also known as MAPRE3) in CHO-K1 cells. These cells are particularly suitable for tracking EB-plus-tip proteins because the MTs display very homogenous growth rates when they grow from the centrosome towards the periphery (Komarova et al., 2009). To ensure the validity of our data and the subsequent conclusions drawn, we specifically analysed cells that expressed the various constructs (wild-type G2L constructs versus mutant G2L constructs) at very similar levels (assessed by fluorescence intensity), enabling us to make valid comparisons between conditions. The effect of both G2L1 and G2L2 expression was dramatic. In cells expressing intermediate to high levels of these proteins, EB3–tdTomato appeared immobilised on actin stress fibres (supplementary material Fig. 1A). Even in cells expressing barely detectable levels of G2L1– or G2L2–GFP (as assessed by fluorescence intensity levels), the dramatic impact on EB protein localisation was apparent. In control cells expressing GFP with EB3–tdTomato, recordings showed that MT plus-ends grew in a random orientation in cells, whereas MT plus-ends grew more slowly and often appeared to tether along actin stress fibres in cells expressing low levels of G2L1 (supplementary material Movie 2). Interestingly, although G2L1 and G2L2 appeared to dramatically affect EB protein localisation and MT growth rates, G2L3 expression had little, if any, effect even when expressed at higher levels (data not shown).

**Fig. 3.**
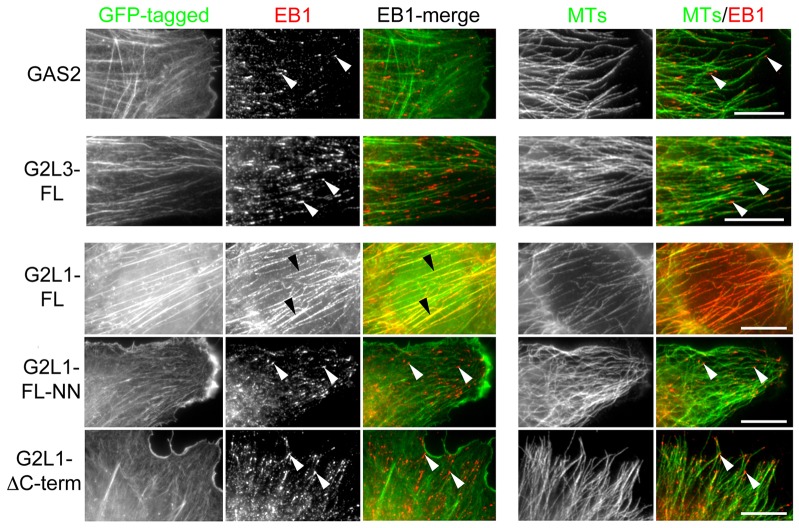
**G2L1 recruits EB proteins along the actin cytoskeleton.** NIH3T3 cells expressing GAS2, G2L3 or G2L1 were fixed and stained for EB1 and MTs. Note the G2L1 colocalisation with EB1 at the actin cytoskeleton (black arrowheads). MtLS mutations (FL-NN) or deletion of the C-terminus (ΔC-term) restore EB1 localisation to MT plus-ends (white arrowheads). GAS2 and G2L3 do not affect the localisation of EB1 (white arrowheads). Scale bars: 5 µm.

Because of the dramatic extent of EB3–tdTomato colocalisation with G2L1 and G2L2, we wondered whether the overexpression of EB proteins would give similar results. We therefore assessed endogenous EB protein localisation using antibodies in cells expressing G2L proteins. In agreement with the above data, both full-length G2L1 and G2L2 recruited endogenous EB1 to actin stress fibres, whereas G2L3 and GAS2 had no effect on EB1 recruitment (Fig. 3; supplementary material Fig. S1B). In order to verify that this effect was mediated through direct interactions between EB proteins and G2L1 or G2L2, we expressed either the EB-protein-binding defective mutants (G2L1-FL-NN and G2L2-FL-NN, respectively) or versions of G2L1 and G2L2 lacking C-termini (G2L1-ΔC-term and G2L2-ΔC-term, respectively). We found that these mutants fully blocked the G2L-mediated EB recruitment and their effect on MT dynamics was lost (Fig. 3; supplementary material Fig. S1A,B).

Previous observations have shown that the expression of G2L C-termini alone are able to induce MT bundling (Goriounov et al., 2003; Stroud et al., 2011), which suggests that their expression alone could affect MT dynamics. However, only the expression of G2L1-C-term led to a very small but significant reduction of MT growth rate as measured by tracking of co-expressed EB3–tdTomato; this was fully rescued upon mutation of its EB-protein-binding site. None of the other C-terminal G2L constructs showed any significant effect on the MT plus-end growth rate (supplementary material Fig. S1C).

Taken together, these data indicate that G2L1 and G2L2 are able to recruit EB proteins to actin stress fibres, where they might guide MT growth along actin. The observations that G2L3 expression had little impact on both EB recruitment and MT dynamics, suggests that this family member has a different function.

### G2L1 and G2L2 regulate microtubule dynamics through their interaction with EBs

**Fig. 4.**
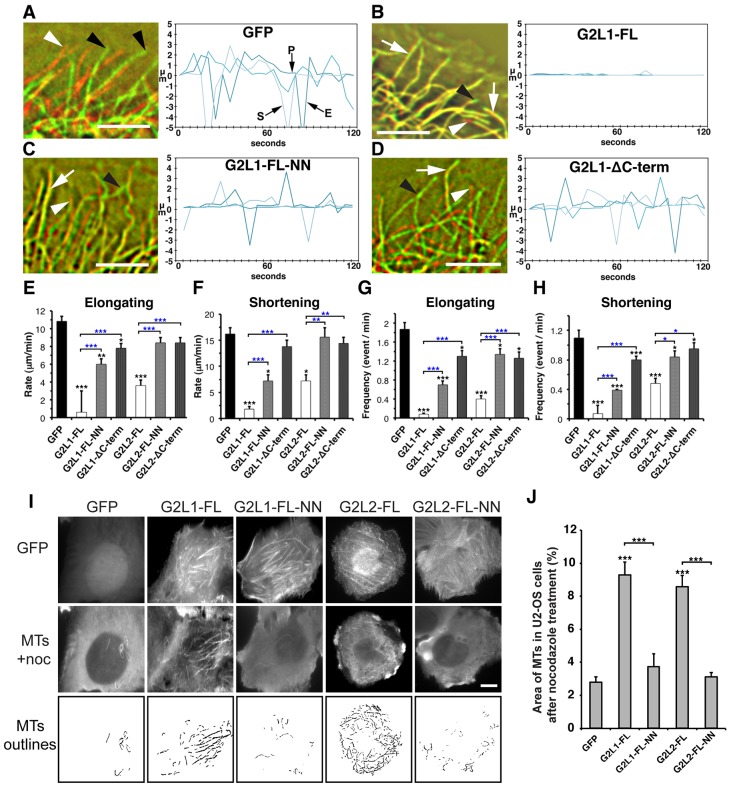
**G2L1 and G2L2 regulate MT dynamics and stabilise MTs through their interaction with EBs.** (A–D) To visualise MT dynamics the first and the last image of a 2-min time-lapse recording of mCherry–tubulin-expressing U2OS cells co-expressing the indicated constructs were labelled in red and green, respectively, and then superimposed. Structures that remained at the same place during the recording appear yellow (white arrows); elongation events appear green (black arrowheads) and shortening events are red (white arrowheads). Note that MT elongation and shortening events are strongly reduced upon co-expression of G2L1-FL, whereas co-expression of G2L1-FL-NN or G2L1-ΔC-term had only minor effects on MT dynamics. MT-lifetime history plots from three representative MTs outline shortening (S), elongating (E), and pausing (P) events of MTs in the GFP control (A). Scale bars: 5 µm. (E,F) Quantification of MT elongation and shortening rates upon expression of indicated G2L1, and G2L2 constructs. (G,H) Frequency of MT elongation and shortening events for the indicated G2L1 and G2L2 constructs. Note that expression of G2L1, and G2L2 significantly decreased the rate and frequency of both shortening and elongation. Blocking the interaction between of G2L1 and G2L2 with EBs through MtLS mutations or C-terminal deletions rescued MT dynamics to a great extent. Results are mean±s.e.m. *P<0.05; **P<0.01; ***P<0.001 (black asterisks indicate significance according to the Student's *t*-test when compared to GFP; blue asterisks indicate significance according to the Student's *t*-test between the indicated constructs). (I) U2OS cells expressing the indicated G2L constructs were treated with nocodazole, then fixed and stained for MTs. Note the increase of nocodazole-resistant MTs in cells expressing G2L1-FL and G2L2-FL compared to their respective EB-binding defective mutants and GFP control. Scale bar: 10 µm. (J) Graph represents quantification of I, where the percentage of the cell area containing MTs was measured in cells expressing G2L constructs after nocodazole treatment.

In the previous experiments we measured MT behaviour indirectly by analysing localisation and dynamics of EB proteins. To measure the direct impact of G2L1 and G2L2 expression on MT dynamics we expressed G2L proteins together with mCherry–tubulin in U2OS cells. These cells displayed a less dense MT network than other cells and therefore were ideal for the analysis of MT dynamics at the cell periphery. A hallmark of MT dynamics is their dynamic oscillation, with phases of growth and shrinkage (Kirschner and Mitchison, 1986). Such dynamic instability is essential for normal cellular function and parameters commonly measured include the rates and frequencies of elongation (E) and shortening (S) (Fig. 4A) (Kirschner and Mitchison, 1986; Desai and Mitchison, 1997).

We observed that expression of GAS2 (supplementary material Fig. S2D) and G2L3 (data not shown) had no effect; however, expression of G2L1 and G2L2 dramatically decreased MT dynamics by significantly increasing the times MTs spent pausing, and decreasing the rates of elongation and shortening (Fig. 4B,E,F; supplementary material Fig. S2). Overall, G2L1 had the most pronounced effect by strongly reducing the oscillatory behaviour of MTs (Fig. 4B). To investigate the role of G2L C-termini and binding of EB proteins on MT dynamics, we expressed the G2L1 and G2L2 mutants in cells. Both of the G2L constructs lacking their respective C-termini (ΔC-term) had minor effects on MT dynamics (Fig. 4D). MT dynamics were almost fully restored in cells with G2L2-NN (Fig. 4C), and partially rescued in cells expressing G2L1-NN (Fig. 4E–H; supplementary material Fig. S2).

A previous study has shown that the expression of G2L1 and G2L2 increased the number of stable MTs that are resistant to the MT depolymerising reagent nocodazole (Goriounov et al., 2003). To test whether this stability was conferred by their interactions with EB proteins, we treated cells expressing the wild-type and mutant G2L proteins with nocodazole for 10, 20, or 30 min prior to fixation, and subsequently stained for MTs. No MTs remained in cells treated for 20 or 30 min with nocodazole (data not shown). However, when cells were treated for 10 min, we observed a more than twofold increase in the amount of stabilised MTs in cells expressing G2L1 and G2L2, compared to their respective EB-binding-defective mutants or GFP alone (Fig. 4I,J). Overall, these data demonstrate that EB binding to the C-termini of G2L1 and G2L2 plays an important role in their ability to regulate MT dynamics and contributes to MT stability.

### G2L1 and G2L2 localisation to actin stress fibres guides MTs in an EB-dependent manner

**Fig. 5.**
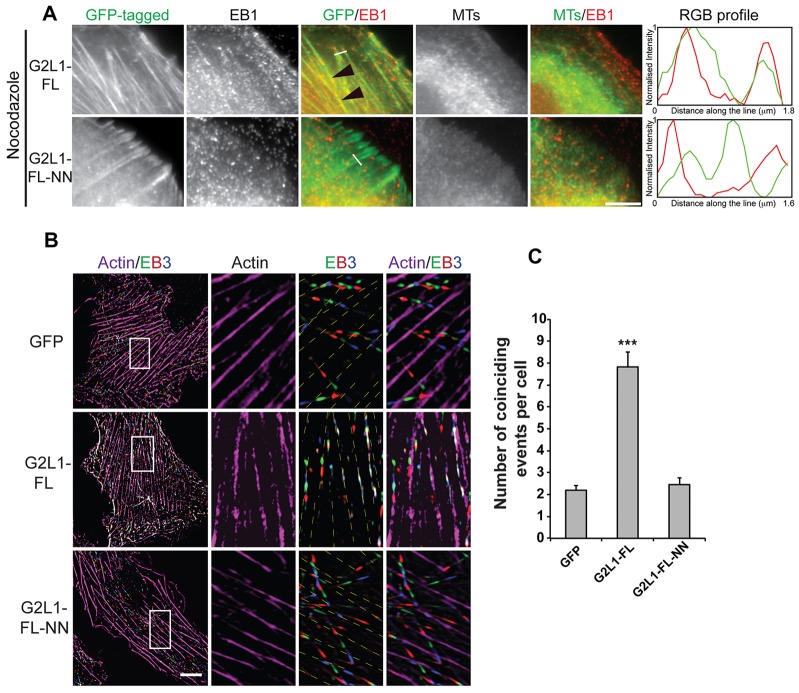
**G2L1 localising on actin stress fibres guides MTs in an EB-dependent manner.** U2OS cells expressing the indicated constructs were treated with nocodazole (A). Note that in contrast to G2L1-FL-NN, G2L1 was able to retain EB1 at actin stress fibres independently of MTs (black arrowheads). Scale bar: 5 µm. The white line indicates the position of the RGB profile shown to the right. (B) Still frames from movies taken of NIH 3T3 cells expressing G2L1-FL or G2L1-FL-NN or GFP together with EB3 (green, red, blue) and actin (magenta). Magnifications indicate localisation of EB3 (merge of three consecutive time frames taken every 5 s) and actin stress fibres. Note that EB3 was guided along actin stress fibres in G2L1-FL-transfected cells. This phenomenon was no longer observed in cells expressing the EB-binding-defective mutant G2L1-FL-NN. Scale bar: 10 µm. (C) Graph represents quantification of B where co-alignment of three consecutive time frames of EB3 along actin stress fibres was considered as one coincidence event.

Because G2L1, and to a lesser extent G2L2, primarily decorate actin stress fibres, we hypothesised that they were actively involved in capturing passing MTs, leading to their co-alignment with stress fibres. To examine possible modes of MT capture, we expressed G2L1 or G2L2 in U2OS cells, then trypsinised and replated them in medium containing nocodazole. Upon spreading, cells were fixed and examined for G2L- and EB protein localisation. Despite the absence of MTs, EB proteins decorated actin stress fibres along with G2L proteins at comparable levels to the control (Fig. 5A; supplementary material Fig. S3A,B). This was completely abolished in cells expressing G2L proteins bearing mutations for EB protein binding.

These observations suggested that G2L1 and G2L2 proteins might be able to capture EB proteins, and that this complex in turn contributes to the regulation of MT dynamics and guidance along actin stress fibres. To address this, we expressed low levels of G2L1 and G2L2 together with actin–CFP and EB3–tdTomato and analysed the cells using time-lapse imaging. We selected three consecutive time points from EB3 recordings, merged them to visualise their history, and analysed how many of the EB-triplicates co-aligned with actin stress fibres. Our data demonstrated that the number of coincidences of co-alignment between actin stress fibres and EB3 tracks was threefold higher in cells expressing G2L1-FL than in control cells expressing GFP or G2L1 deficient in EB binding (G2L1-FL-NN) (Fig. 5B,C). In contrast to G2L1, we were unable to quantify co-alignment for G2L2 because EB3 localisation appeared to be static, even in cells expressing very low levels of G2L2. EB3 localisation was fully restored to growing MT plus-ends in cells expressing G2L2-FL-NN, thereby confirming that the crosstalk between actin and MT mediated by G2L2 is primarily coordinated by its ability to bind EB proteins.

### The degree of actin–microtubule crosstalk mediated by G2L1 and G2L2 is different in cells plated on fibronectin and Cell-Tak

**Fig. 6.**
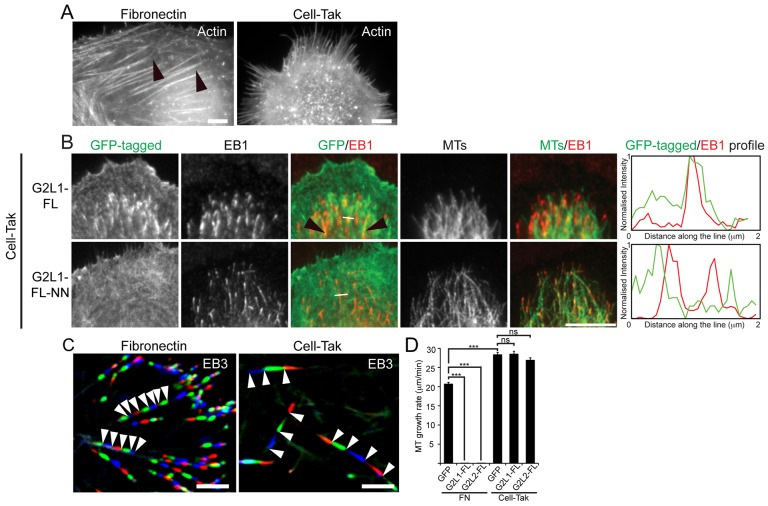
**The effect of G2L proteins on MT dynamics is dependent on the extracellular matrix.** (A) CHO-K1 cells expressing GFP were plated on either fibronectin (left panel) or Cell-Tak (right panel), and actin was visualised with either phalloidin or Lifeact–RFP. Note the absence of prominent actin stress fibres in cells plated on Cell-Tak in comparison to fibronectin (black arrowheads). (B) U2OS cells expressing the indicated constructs plated on Cell-Tak. Note that G2L1 was present on fine actin structures, but predominantly localised to EB1-positive MT plus-ends (black arrowheads), whereas the EB-binding-defective mutant (G2L1-FL-NN) predominantly localised to actin. White lines indicate regions taken for intensity profile measurements shown to the right. (C) CHO-K1 cells expressing EB3–tdTomato were plated either on fibronectin or Cell-Tak and time-lapse images were recorded in 5-s intervals. Consecutive frames of EB3 displayed in red, green and blue, were superimposed to visualise MT growth over time. The decrease in distance between red, green and blue MT tips in cells plated on fibronectin versus cells on Cell-Tak outlines the impact of actin organisation on MT dynamics (white arrowheads). (D) Quantification of MT growth speed measured by plus-end-tip displacement of cells expressing indicated constructs (*n* = 165 MT plus-ends from >5 cells per condition). Note the reduction of MT growth rates on fibronectin (FN) and the failure of G2L1 and G2L2 to stabilise MT when plated on Cell-Tak. ***P<0.001; ns, not significant (*P*>0.1), according to the Student's *t*-test between indicated constructs. Scale bars: 5 µm.

From the above observations, we hypothesised that cells with a less-pronounced actin cytoskeleton would be less able to affect MT growth rates. To test this we plated cells on Cell-Tak, a reagent that promotes cell spreading without engaging integrins. Cells on Cell-Tak lack focal adhesions, and consequently have vastly reduced numbers of actin stress fibres (Fig. 6A). Under these conditions, G2L1 and G2L2 still localised to the fine actin meshwork, but showed a more pronounced colocalisation with EB-enriched MT tips (Fig. 6B). The presence of EB1 itself at the tip of MTs indicated that these MTs are dynamic. To visualise MT growth under these conditions we expressed EB3–tdTomato together with G2L1 or G2L2 in CHO-K1 cells and plated them on fibronectin or Cell-Tak. In agreement with our data in Fig. 4, cells expressing intermediate levels of G2L1 or G2L2 that were plated on fibronectin displayed significantly attenuated MT plus-end growth rates compared to the GFP control. In contrast, MT growth rates of cells on Cell-Tak were unaffected, despite them expressing similar levels of G2L proteins (as assessed by fluorescence intensity), compared to GFP-expressing control cells (Fig. 6C,D; supplementary material Movies 3, 4).

These experiments demonstrate that G2L1 and G2L2 support efficient crosstalk between actin and MTs, and that the impact of this crosstalk on MT dynamics greatly varies depending on the presence of extracellular matrix.

## DISCUSSION

Previous publications have outlined the potential of GAS2 family members being involved in crosslinking between actin and MTs (Goriounov et al., 2003; Stroud et al., 2011). From these publications, it seemed clear that the binding of these proteins to actin stress fibres was mediated through their N-terminal CH domains, and the binding to MTs was mediated by their C-termini, which vary between the different members. In this study, we first compared four GAS2 proteins and analysed their roles in regulating the crosstalk between MTs and F-actin. We identified classical EB-binding motifs in the C-termini of two out of the four members, and demonstrated that the ability of the different family members to mediate actin–MT crosstalk strongly depended on the presence of these binding motifs.

### Role of G2L association with EBs and relationship with spectraplakins

Although the mode and role of EB protein binding to G2L3 needs further investigation, our mutational analysis showed that EB protein binding is the main mechanism by which G2L1 and G2L2 regulate communication with MTs. The interaction with EB proteins was not only responsible for MT guidance along actin fibres, but also determined the state of MT dynamics and their stability. This interaction with EBs and their role in MT guidance places G2L1 and G2L2 in a close functional relationship with spectraplakins (Alves-Silva et al., 2012). To what extent the function of mammalian spectraplakins, MACF1, dystonin (human) and ACF7 (mouse), in guiding MTs along actin stress fibres is dependent on their C-terminal EB-binding sites has not been explored in detail (Leung et al., 1999; Kodama et al., 2003; Slep et al., 2005; Honnappa et al., 2009). However, it is likely that they have similarly important roles to those of G2L1 or G2L2, because it has been shown that the MtLS in the *Drosophila* spectraplakin homologue Short stop (Shot) is important for its localisation to growing MT tips (Applewhite et al., 2010) and essential for its physiological function in axonal outgrowth (Alves-Silva et al., 2012).

Like spectraplakins, EB binding seems not to be the sole link between G2L proteins and MTs (Leung et al., 1999; Goriounov et al., 2003), especially because isolated C-terminal ends of G2L1 and G2L2 with mutated MtLS still localise (albeit weakly) to the MT lattice. We speculate that such interactions might be mediated through the positive charges in the G2L C-termini, which have been shown previously to enable electrostatic peptide interactions with MTs [which have an overall negative charge (Wolff, 1998)]. In support of this, positively charged residues in the C-terminus of Shot were found to be important for both MT shaft and plus-end localisation (Alves-Silva et al., 2012). What precise role such interactions might have for the function of G2L proteins remains to be determined, but they might be important to stabilise MTs.

### G2L proteins and the regulation of MT dynamics versus stability

Our data clearly show that members of the G2L proteins regulate MT dynamics, which is dependent on the presence of EB-binding sites, and that G2L proteins promote MT stability (measured by nocodazole resistance). Increased MT stability was limited to short-term drug treatment (10 min) and was almost completely abolished in MtLS mutants, demonstrating that EB binding is an important modulator of G2L-induced MT stability. EB binding, however, is not likely to be the only stabilising factor for G2L-mediated MT stability, because Goriounov and colleagues have found that constructs containing the GAR domain and C-terminus also contribute to nocodazole resistance (Goriounov et al., 2003). Similar experiments with the *Drosophila* spectraplakin Shot have shown a role for the GAR domain in MT stabilisation (Alves-Silva et al., 2012) suggesting contributions from both the C-terminal tail and the GAR domain.

### Cellular and physiological roles of GAS2 family members

The physiological role of GAS2 family members in mammals is unclear, but clues about their function have been revealed in *Drosophila*, which express only a single member of the GAS2 family, named ‘Pigs’. Pigs-null *Drosophila* are semi-viable, and display muscle degeneration and defects in follicle cell differentiation (Pines et al., 2010). The C-terminus of Pigs has been found to be important for its correct localisation in *Drosophila* cells, but the role of the four putative MtLS motifs in its C-terminus has yet to be determined.

**Fig. 7.**
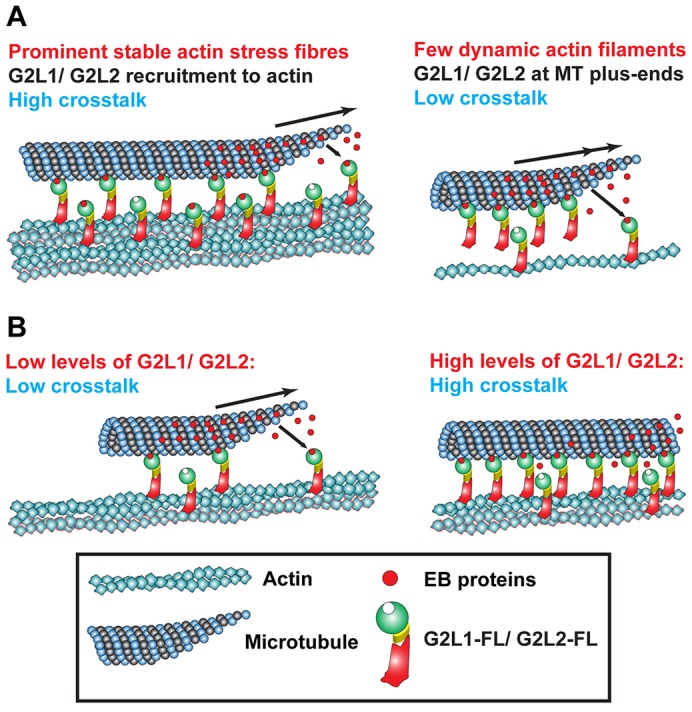
**Model of G2L-mediated crosstalk.** (A) G2L1 and G2L2 act as sensors and detect actin structures that require crosstalk with MTs. (B) Changes in requirements of crosstalk in the cell might be regulated either by modulating their endogenous expression levels or by changing the binding strength to actin or EBs.

In mammals, it appears that GAS2 family members have diverse cellular functions despite their highly similar domain organisation. Although GAS2 seems to be involved in the regulation of apoptosis (Schneider et al., 1988; Brancolini et al., 1995), G2L3 seems to be involved in mitosis (Wolter et al., 2012). Cellular and physiological functions of G2L1 and G2L2 are less established (Gamper et al., 2009), but our data suggest that their functions are based on their strong ability to mediate crosstalk between the actin and MTs through EB proteins. This could be important in growth arrest, when their otherwise very low expression is upregulated (Brancolini et al., 1995), or under circumstances that promote G2L binding to actin. The latter scenario is supported by our observation that the crosstalk between actin and MTs mediated by G2L1 and G2L2 is most pronounced when cells develop stable actin stress fibres (Fig. 1). Table 1 summarises the characteristics and behaviour of the GAS2 family proteins. From these data, we propose a model whereby G2L1 and G2L2, which have a high affinity to actin, bind to cytoplasmic EB proteins that might, in turn, associate with MTs. Alternatively, G2L1 and G2L2 might capture MT tips enriched in EB proteins, thus guiding them along actin stress fibres (Fig. 7A). Hence, G2L1 and G2L2 sense the state of actin structures that can change under different environmental conditions. A further possibility to modulate the crosstalk in the cell might occur through the regulation of endogenous G2L protein expression levels (Fig. 7B).

**Table 1.**
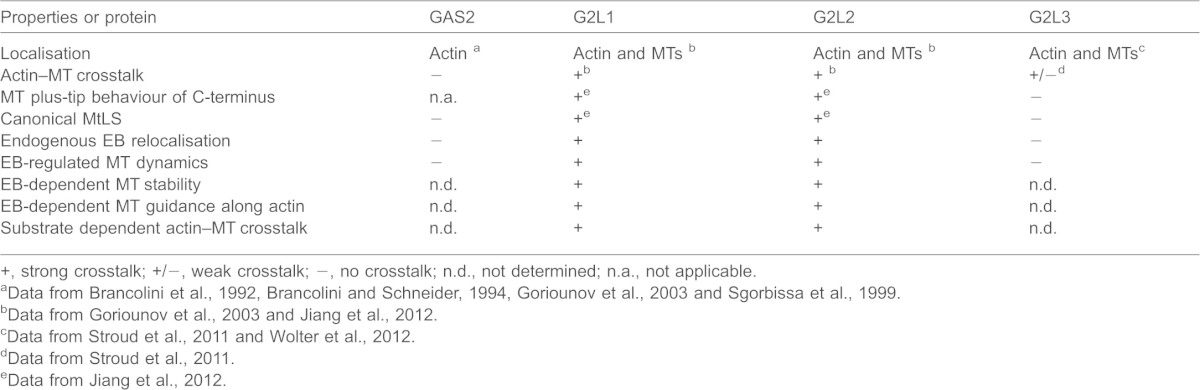
A summary of the characteristics and behaviour of the GAS2 protein family

+ , strong crosstalk; +/−, weak crosstalk; −, no crosstalk; n.d., not determined; n.a., not applicable.

a Data from Brancolini et al., 1992, Brancolini and Schneider, 1994, Goriounov et al., 2003 and Sgorbissa et al., 1999.

b Data from Goriounov et al., 2003 and Jiang et al., 2012.

c Data from Stroud et al., 2011 and Wolter et al., 2012.

d Data from Stroud et al., 2011.

e Data from Jiang et al., 2012.

## MATERIALS AND METHODS

### Cell culture and transfections

All cells were grown in DMEM (Sigma Aldrich, Dorset, UK) supplemented with 10% FBS, and 1% glutamine in a 5% CO_2_ humidified incubator. For transfections of CHO-K1, NIH3T3 and COS-1, cells were plated in six-well dishes (for immunofluorescence) or 10-cm Petri dishes (for EB1–GST pull-down experiments) using 1–1.5 µg or 8 µg of DNA, respectively, and Lipofectamine Plus (Invitrogen, Paisley, UK) in accordance with the manufacturer's instructions. U2OS cells were transfected with Lipofectamine 2000 (Invitrogen, Paisley, UK) with 1–1.5 µg of DNA. For immunofluorescence, cells were replated after 3–4 h in glass-bottomed dishes (MatTek Corporation, Ashland, USA) coated with 10 µg/ml bovine fibronectin (pFN; Sigma) or 3.5 mg/cm^2^ Cell-Tak (Becton, Dickinson Biosciences, Oxford, UK). For pulldown experiments, cells were washed three times in PBS (Lonza, Verviers, Belgium), and once in complete DMEM, and left overnight before lysis the following day.

### Fixed immunofluorescence imaging

Cells were fixed and permeabilised with 3% paraformaldehyde (Sigma) containing 0.25% Triton X-100 (Sigma) and 0.05% glutaraldehyde (Sigma) for 15 min, before being washed in PBS (Lonza). Autofluorescence was quenched using 0.01% sodium tetrahydroborate (Sigma) in PBS. Cells were incubated with anti-tubulin (DM1A, Sigma) antibody, at 1∶500 dilution, followed by secondary antibodies conjugated to DyLight 488, 594 or 649 (Jackson ImmunoResearch Laboratories, Suffolk, UK). For actin, Texas-Red-, FITC- or Alexa-Fluor-633-labelled phalloidin (Invitrogen) was added together with the secondary antibody. For the EB1 antibody (1A11/4, Santa Cruz Biotechnology), cells were fixed in −20°C methanol for 5 min, before being rehydrated in PBS and stained using anti-tubulin antibodies (as above). For the experiments involving nocodazole (Sigma) and Cell-Tak, cells were spread on glass-bottomed dishes in the presence of 10 or 4 µM nocodazole or DMSO (control) and Cell-Tak, respectively, for 1 h prior to fixation. Cells were then imaged using an oil-immersed 100× objective, with 1.35 numerical aperture, on an inverted microscope (IX71; Olympus) controlled by a Deltavision system (Applied Precision, Washington, USA). Images were captured using a Coolsnap HQ CCD camera (Princeton Instruments, Lurgan, UK).

### Live-cell imaging

For MT dynamics and EB-tracking experiments, cells were plated on glass-bottomed dishes coated with fibronectin or Cell-Tak and spread for 1 h prior to live imaging. Cells were imaged in Ham's F12 medium supplemented with 25 mM HEPES, 1% L-glutamine, 1% penicillin/streptomycin and the pH was adjusted to 7.3 with NaOH (as described in Carisey et al., 2011) either using an oil-immersed 60× objective, with 1.35 numerical aperture, on a Yokogawa spinning disk microscope (Intelligent Imaging Innovations, Colorado, US) equipped with an Evolve EMCCD camera (Photometrics, AZ, USA) or an oil-immersed 100× objective, with 1.35 numerical aperture, on an inverted microscope (IX71; Olympus) controlled by a Deltavision system (Applied Precision). Images were captured using a Coolsnap HQ CCD camera (Princeton Instruments).

### Image processing

To measure MT plus-end growth rates, automated tracking software was used (Matov et al., 2010), Briefly, cells co-expressing EB3–tdTomato and G2L C-termini were imaged every 2 s and the default settings were used for analysis. Analysis of MT dynamics were performed as described previously (Dhamodharan and Wadsworth, 1995), with some modifications. Briefly, to assess rates and frequencies of MT elongation, shortening and pausing, cells were imaged every 5 s. A pause phase was determined when MT movement was not greater than ±0.5 µm, the shortening phase as MT movement greater than −0.5 µm, and elongation as MT movement greater than +0.5 µm. MT dynamics were measured manually by using the Fiji manual tracking plugin (Schindelin et al., 2012). To measure the percentage of the cell area containing MTs in cells treated with nocodazole, images were processed and analysed with Fiji software. To detect MTs, the image background was subtracted, a region of the cell surface was selected and an FFT bandpass filter and threshold was applied. Coinciding events were measured manually. Briefly, at least 30 cells were imaged every 5 s, RGB merges of three consecutive time frames of EB3–tdTomato and Actin–CFP were prepared using Fiji software. Each co-alignment of three consecutive time frames of EB3 along actin stress fibres was considered as one coincidence event. To measure colocalisation of GAS2 family members and EB proteins, RGB merges were prepared by using Fiji software. MATLAB was used to create fluorescence intensity line profiles over merged images and graphs representing normalised fluorescence intensity over the drawn line.

Adobe Photoshop CS5 and Adobe Illustrator CS4 were used in the preparation of figures for this manuscript.

### Constructs and sub-cloning

For immunofluorescence, G2L3 and G2L3-C-T were cloned into pEGFP-N1 or mCherry-N1 vectors (Clontech) using the restriction endonucleases (Fermentas) NheI and HindIII at the amino acids indicated in Fig. 1A. G2L3-C-T was also cloned into the pEGFP-C1 vector (Clontech) using the restriction endonuclease BamHI. GAS2 was cloned into pEGFP-N1 with XhoI and KpnI, and pEGFP-C1 with EcoRI and BamHI. G2L1-FL and G2L1-C-term were cloned into pEGFP-C1 with EcoRI and BamHI, G2L1-C-term was cloned into pEGFP-N1 with BglII and HindIII. G2L2-FL and G2L2-C-term were cloned in to pEGFP-C1 with BglII and EcoRI, G2L2-C-term was cloned in to pEGFP-N1 with NheI and HindIII. For recombinant expression in *E. coli*, GST–EB1 was cloned into the pHisGST vector (Kammerer et al., 1998) using the restriction endonucleases EcoRI and BamHI. All recombinant insert DNA was verified by DNA sequencing (GATC Biotech). EB1–GFP was a kind gift from the laboratory of Shoichiro Tsukita. EB3–tdTomato construct was a kind gift from the laboratory of Philip Woodman (University of Manchester, UK). The mCherry–tubulin construct was a kind gift from the laboratory of Viki Allan (University of Manchester, UK). Lifeact-RFP construct was a kind gift from the laboratory of Roland Wedlich-Soldner's lab (MPI Munich, Germany).

### Protein expression and purification

The recombinant GST-EB1 was expressed in *E. coli* JM109 (DE3) (Merck Biosciences) and BL21 CodonPlus (DE3) (Stratagene, UK), as previously described (Kammerer et al., 1995) using an autoinduction protocol (as described by Studier, 2005). GST–EB1-C-T was purified using glutathione-coated Sepharose beads (GE Healthcare). Purification was performed under denaturing condition using 8 M urea as described in the pET system manual (Merck Biosciences). Removal of the GST tag by thrombin was performed as previously described (Kammerer et al., 1995). Determination of protein concentration was achieved by measuring the absorbance of tryptophan and tyrosine residues at 280 nm (Edelhoch, 1967).

### GST pull-down assay from cell lysates

COS cells were washed with PBS, trypsinised, centrifuged and lysed in 500 µl ice-cold lysis buffer (1% NP40, 50 mM Tris-HCl pH 7.4, 120 mM NaCl, 2.5 mM EGTA 10 mM MgCl, supplemented with 2× protease inhibitor cocktail solution). Lysates were passed through a 27G needle ten times, centrifuged in a pre-cooled centrifuge at 4°C for 15 min at 800 ***g***. The supernatant was pre-cleared with glutathione-coated Sepharose beads (GE Healthcare) for 1 h at 4°C, followed by centrifugation at 2000 ***g*** and aspiration of the supernatant. Glutathione-coated Sepharose beads were incubated with either GST–EB1 or GST alone (control). After pre-clearing, the GST–EB1 or GST-coated beads were washed three times in PBS containing 0.01% Tween 20 (PBS-T), and were resuspended in the pre-cleared lysate and rotated for an hour at 4°C. The samples were centrifuged at 2000 ***g***. The beads were washed four times in PBS-T and the bound proteins were eluted from the beads in 2× SDS-loading buffer. The individual fractions were then separated by SDS-PAGE and analysed by immunoblotting, using an anti-GFP antibody (1∶1000, Roche).

### Statistical analysis

Student's *t*-tests were performed using Microsoft Excel.

## Supplementary Material

Supplementary Material
