# Supplementary material for: GAS2-like proteins mediate communication between microtubules and actin through interactions with end-binding proteins
Source: J Cell Sci. 2014 Jun 15;127(12):2672–82. doi: 10.1242/jcs.140558 (PMC4058111; doi:10.1242/jcs.140558)
Supplement: Supplementary Material [file supp_127_12_2672__index.html]

GAS2-like proteins mediate communication between microtubules and actin through interactions with end-binding proteins — Supplementary Material 

# GAS2-like proteins mediate communication between microtubules and actin through interactions with end-binding proteins

## JCS140558 Supplementary Material

**Files in this Data Supplement:**

- **Supplementary Material**
