# Supplementary material for: GAS2-like proteins mediate communication between microtubules and actin through interactions with end-binding proteins
Source: J Cell Sci. 2014 Jun 15;127(12):2672–82. doi: 10.1242/jcs.140558 (PMC4058111; doi:10.1242/jcs.140558)
Supplement: Supplementary Material [file supp_127.12.2672_JCS140558.pdf]

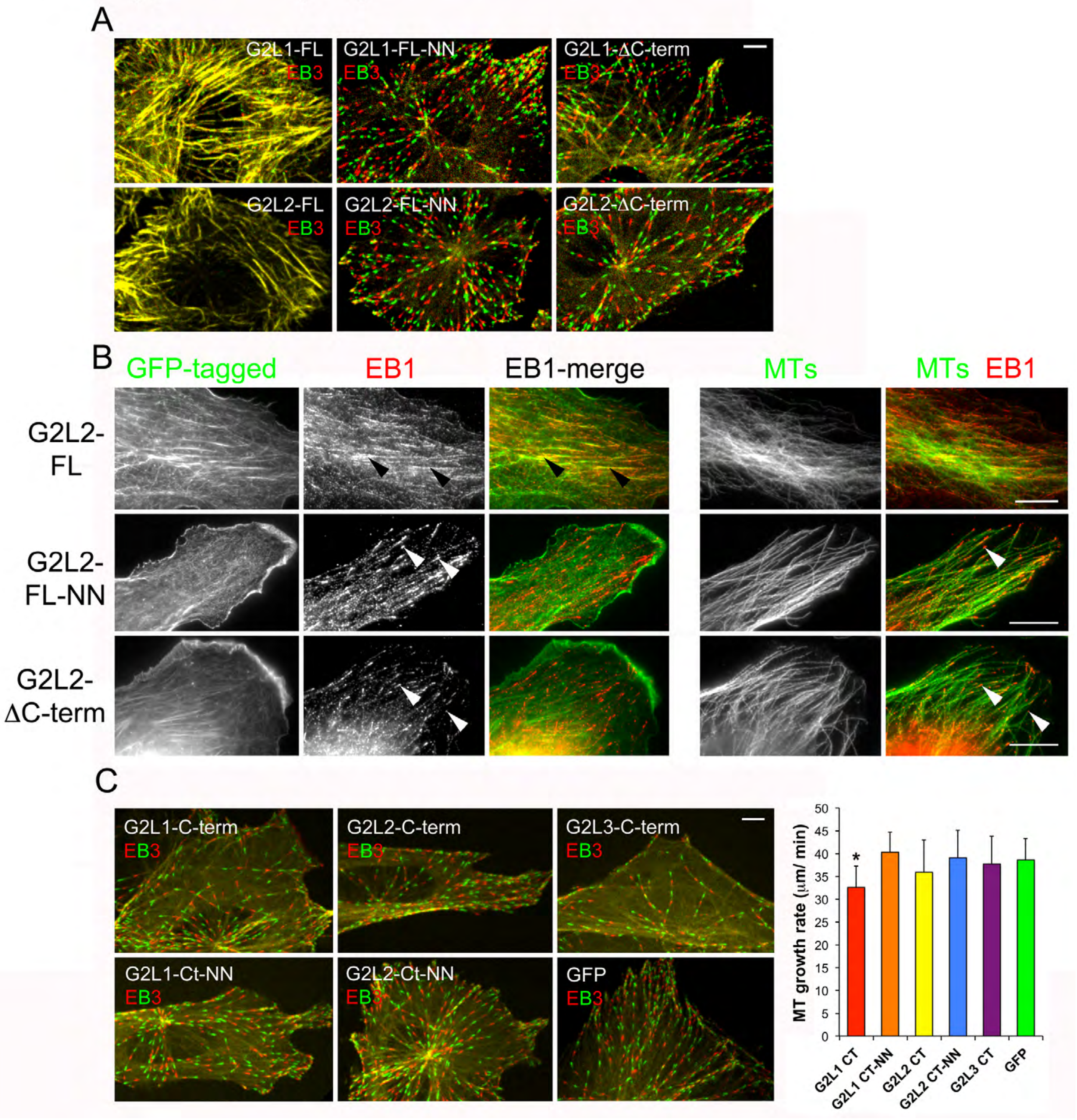

Fig. S1. **(A)** CHO-K1 cells co-expressing EB3 and full-length G2L1 and G2L2 and their respective NN and ΔC-term mutants were imaged live at 5 seconds intervals. Consecutive frames of EB3 were imaged in red and green and superimposed on one another. Note the large overlap between red and green channels from consecutive frames in the wild-type (G2L1-FL and G2L2-FL) panel, outlining the static nature of EB3 in these cells. Conversely, preventing EB3 binding to G2L1 and G2L2 by MtLS mutations or by deletion of C-termini, rescued EB3 MT plus-end tip-tracking. **(B)** NIH 3T3 cells expressing the full-length G2L2 were fixed and stained for EB1 and MTs. Note G2L2 colocalisation with EB1 at the actin cytoskeleton (black arrowheads). MtLSs mutations (FL-NN) or removal of the C-terminus (ΔC-term) restored EB1 localisation to growing MT plus-ends (white arrowheads). **(C)** CHO-K1 cells co-expressing EB3 and GAS2-like C-termini and their respective NN mutants were recorded live at 5 seconds intervals and imaged as in (A). Average speeds of MT plus-ends are indicated in the histogram. More than 4000 MT plus-ends were tracked for each condition. Error bars indicate standard deviations from the mean. Black asterisks indicates $p<0.005$ according to the Student's T-test. Scale bars, 5mm.

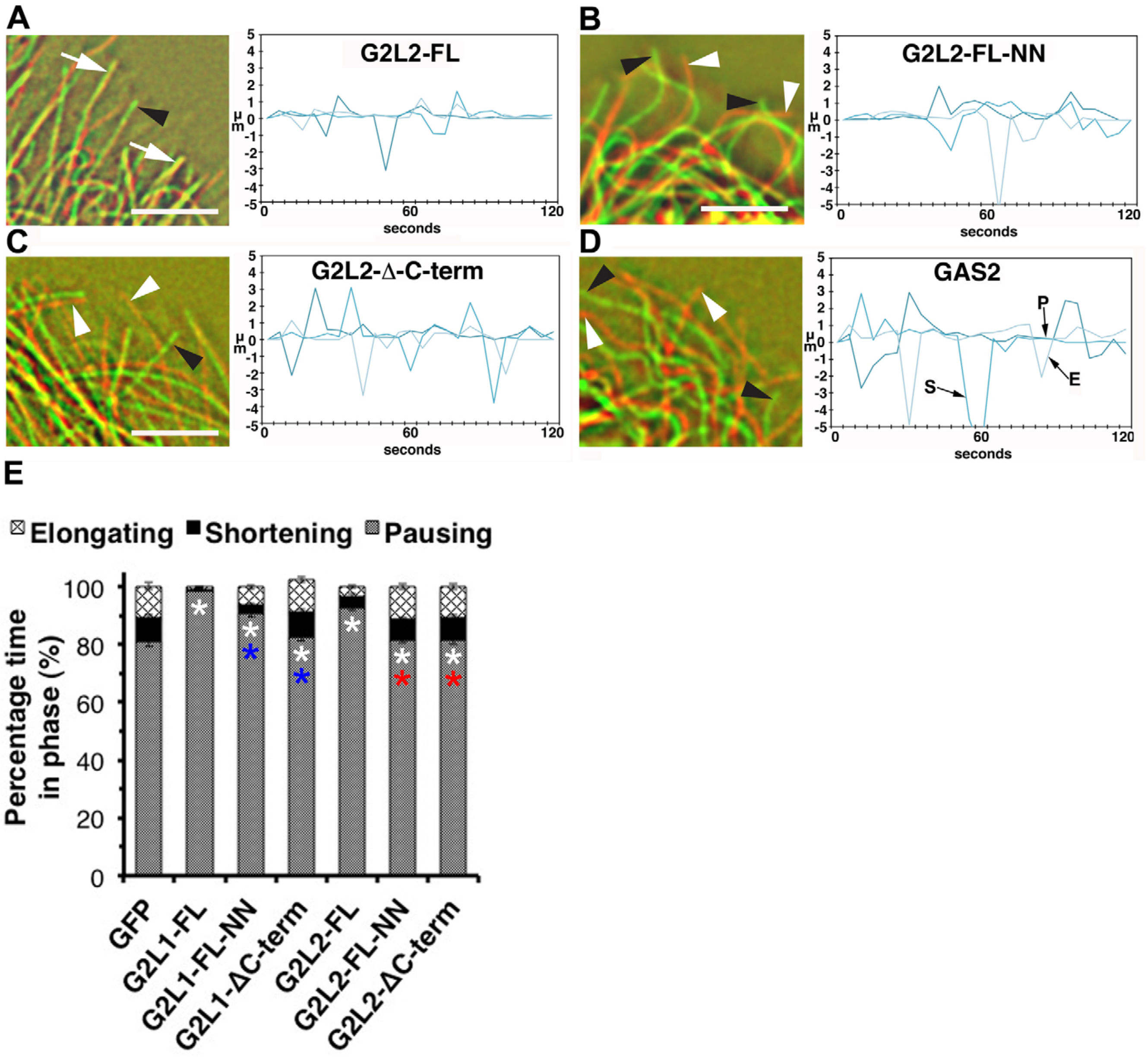

Fig. S2. Micrographs from panels A-D. Overlays of the first frame (MTs in red) and the last frame at 2 min (MTs in green) from time-lapse imaging of MTs in U2OS cells expressing the indicated constructs of G2L2 **(A-C)**, or GFP **(D)** (control). White arrowheads outline MTs that have undergone shortening; black arrowheads outline newly polymerised MTs; and white arrows indicate paused MTs. Note that the expression of G2L2-FL dramatically attenuates MT dynamics. MT lifetime-history plots of three representative MTs from their respective micrographs to the left. The shortening (S), elongating (E) and pausing (P) phases of MT dynamic instability are denoted in the GFP control **(D)**. Note that MTs in cells expressing G2L2-FL exhibit very little dynamic instability in comparison to the GFP-expressing control. **(E)** Percentage time spent by MTs in each phase of dynamic instability for the indicated G2L1 and G2L2 constructs, respectively. Note the amount of time MTs spent in pause is significantly increased in cells expressing G2L1 and G2L2. White asterisks indicate $p<0.05$ according to the Student's T-test compared to GFP, blue and red asterisks indicate $p<0.05$ according to the Student's T-test between the indicated constructs and G2L1-FL and G2L2-FL, respectively. Scale bars, 5 μm.

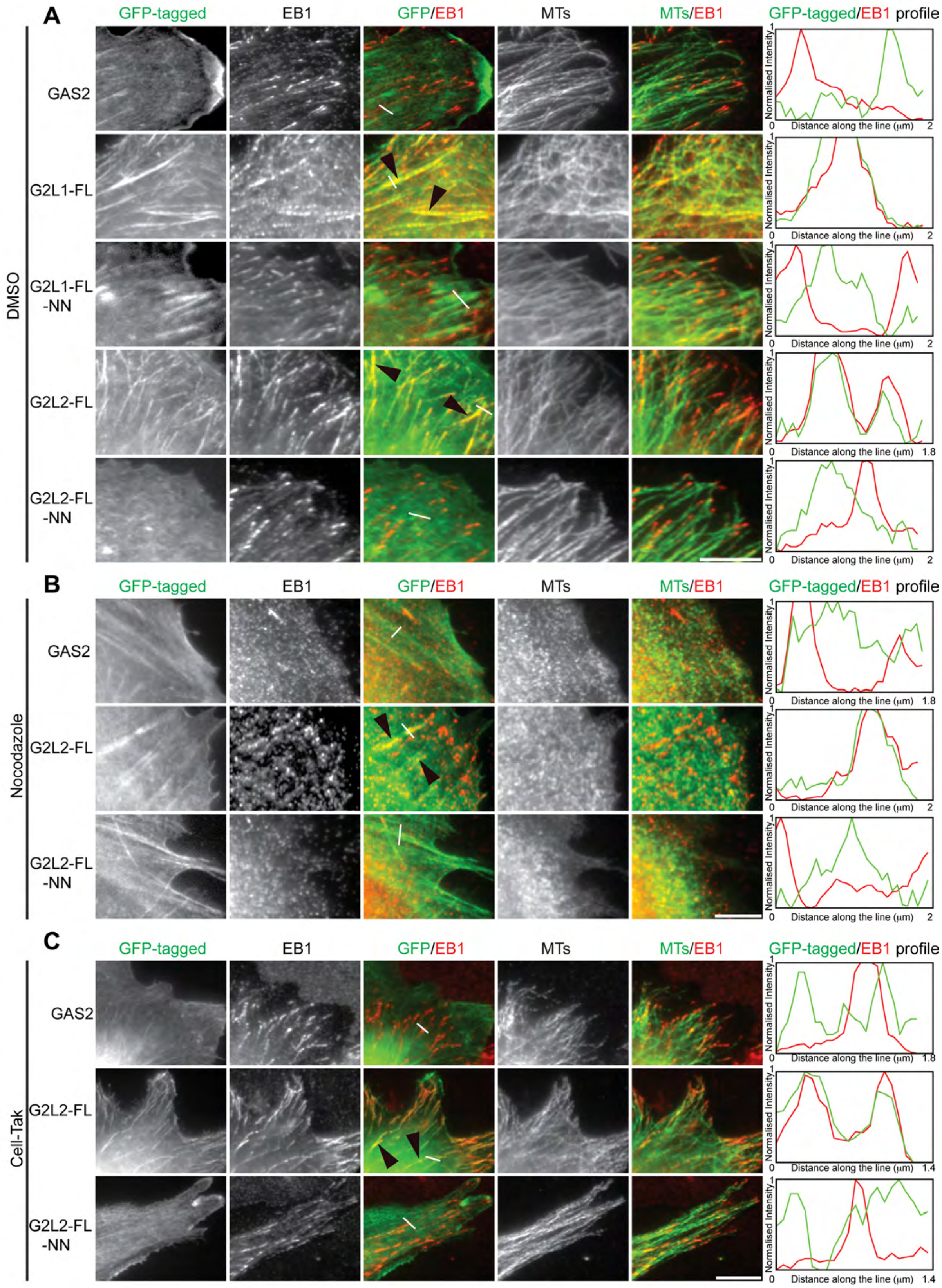

Fig. S3. U2OS cells expressing the indicated constructs were treated with DMSO (**A**). Note that in contrast to G2L1-FL and G2L2-FL (black arrowheads), GAS2, G2L1-FL-NN and G2L2-FL-NN are unable to recruit EB1 to actin structures. (**B**) U2OS cells expressing the indicated constructs were treated with nocodazole. Note that G2L2-FL was able to retain EB1 at actin stress fibres independently of MTs (black arrowheads), whereas EB1 localises diffusely in cells expressing GAS2 or G2L1-FL-NN. (**C**) Cells expressing the indicated constructs were plated on Cell-Tak. Note that G2L2-FL was present on fine actin structures, but predominantly localised to EB1-positive MT plus-ends (black arrowheads), whereas EB-binding defective mutant G2L2-FL-NN and GAS2 predominantly localised to actin. White lines indicate regions taken for intensity profile measurements to the right. Scale bars, 5 μm.

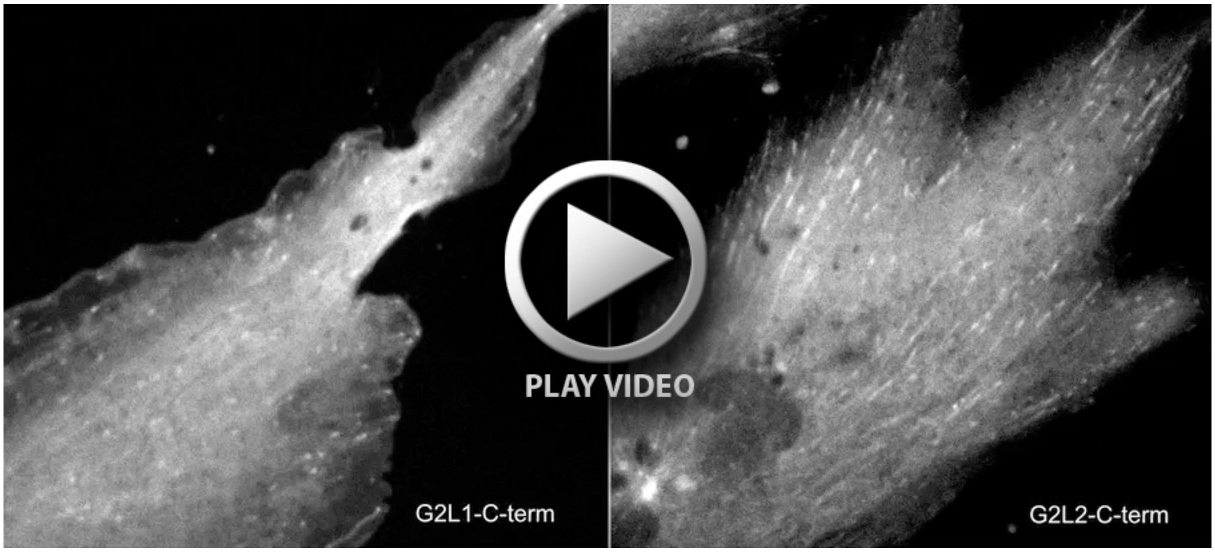

Movie 1. G2L1- and G2L2- C-termini tip track microtubule plus-ends. CHO-K1 cells expressing GFP-conjugated C-termini of either G2L1 or G2L2 were recorded in 5 second intervals for the duration of 60 seconds. Movies are played back at 5 frames per second.

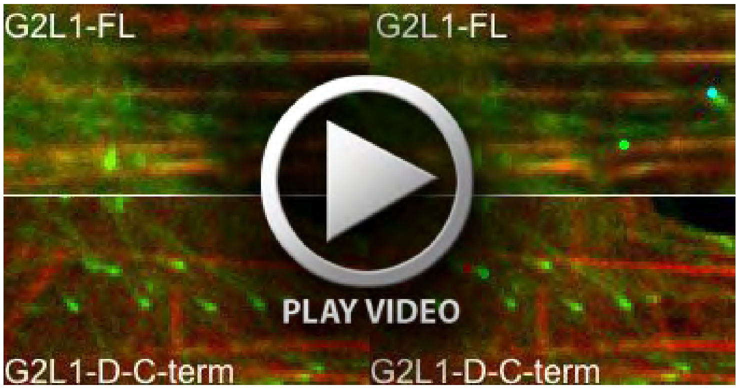

Movie 2. G2L1 requires its C-terminus to guide MTs along actin. Left panels: CHO-K1 cells co-expressing GFP-conjugated G2L1-FL or G2L1-ΔC-term (G2L1-D-C-term) (red) with EB3-tdTomato (green). Right panels show EB3 track overlays for their respective movies in the left panels. Note that for G2L1-FL, EB3 often grows slowly along the G2L1-decorated actin cytoskeleton or dramatically slows down when it contacts actin stress fibres. Conversely, in cells expressing G2L1-ΔC-term, EB3 speeds are unaffected when passing through actin and EB3 is apparently not guided along actin. Cells were imaged live at 2 second intervals for 60 seconds. Movies are played back at 7 frames per second.

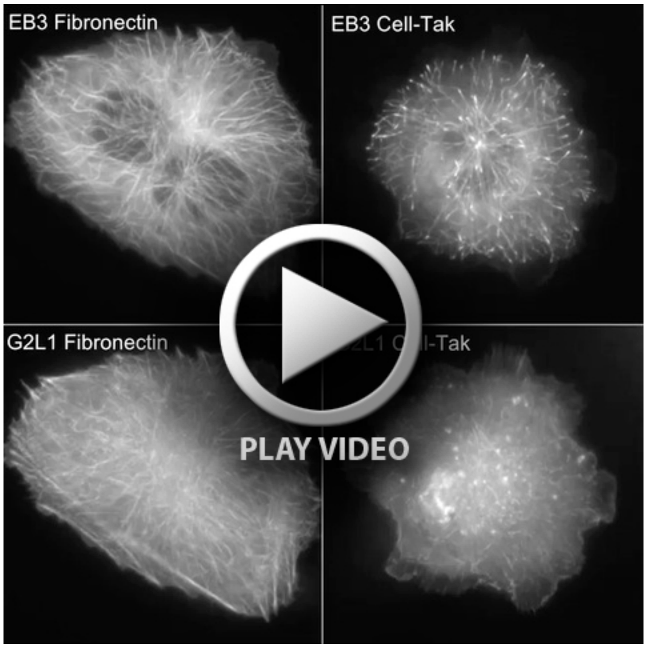

Movie 3. The crosstalk between actin and MTs mediated by G2L1 and EBs is matrix-dependent. CHO-K1 cells co-expressing full-length G2L1 and EB3 plated on FN show high levels of crosstalk between MTs and actin as indicated by the reduced levels of EB3 plus-end tracking. Conversely, very little crosstalk is observed in cells plated on Cell-Tak, which have less prominent actin structures. Selected cells have similar expression levels of both proteins. Cells were recorded every 5 seconds for 120 seconds. Movies are played back at 20 frames per second.

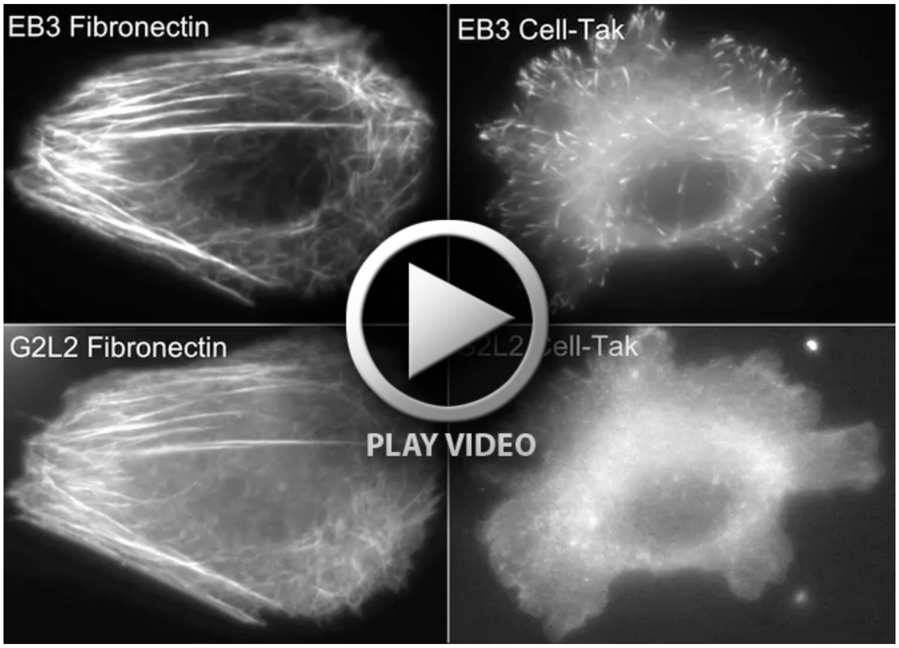

Movie 4. The crosstalk between actin and MTs mediated by G2L2 and EBs is matrix-dependent. CHO-K1 cells co-expressing full-length G2L2 and EB3 plated on fibronectin show high levels of crosstalk between MTs and actin as indicated by the reduced levels of EB3 plus-end tracking. Conversely, very little crosstalk is observed in cells plated on Cell-Tak, which have less prominent actin structures. Selected cells have similar expression levels of both proteins. Cells were recorded every 5 seconds for 120 seconds. Movies are played back at 20 frames per second.
